# Hash-Based Core Genome Multilocus Sequence Typing for Clostridium difficile

**DOI:** 10.1128/JCM.01037-19

**Published:** 2019-12-23

**Authors:** David W. Eyre, Tim E. A. Peto, Derrick W. Crook, A. Sarah Walker, Mark H. Wilcox

**Affiliations:** a Big Data Institute, University of Oxford, Oxford, United Kingdom; b National Institute for Health Research Oxford Biomedical Research Centre, Oxford, United Kingdom; c Nuffield Department of Medicine, University of Oxford, Oxford, United Kingdom; d National Institutes of Health Research Health Protection Unit on Healthcare Associated Infections and Antimicrobial Resistance, University of Oxford, Oxford, United Kingdom; e Healthcare Associated Infections Research Group, University of Leeds, Leeds, United Kingdom; University Hospital Münster

**Keywords:** *Clostridium difficile*, whole-genome sequencing, core genome MLST, quality assurance

## Abstract

Pathogen whole-genome sequencing has huge potential as a tool to better understand infection transmission. However, rapidly identifying closely related genomes among a background of thousands of other genomes is challenging. Here, we describe a refinement to core genome multilocus sequence typing (cgMLST) in which alleles at each gene are reproducibly converted to a unique hash, or short string of letters (hash-cgMLST). This avoids the resource-intensive need for a single centralized database of sequentially numbered alleles. We test the reproducibility and discriminatory power of cgMLST/hash-cgMLST compared to those of mapping-based approaches in Clostridium difficile, using repeated sequencing of the same isolates (replicates) and data from consecutive infection isolates from six English hospitals. Hash-cgMLST provided the same results as standard cgMLST, with minimal performance penalty. Comparing 272 replicate sequence pairs using reference-based mapping, there were 0, 1, or 2 single-nucleotide polymorphisms (SNPs) between 262 (96%), 5 (2%), and 1 (<1%) of the pairs, respectively. Using hash-cgMLST, 218 (80%) of replicate pairs assembled with SPAdes had zero gene differences, and 31 (11%), 5 (2%), and 18 (7%) pairs had 1, 2, and >2 differences, respectively. False gene differences were clustered in specific genes and associated with fragmented assemblies, but were reduced using the SKESA assembler. Considering 412 pairs of infections with ≤2 SNPS, i.e., consistent with recent transmission, 376 (91%) had ≤2 gene differences and 16 (4%) had ≥4. Comparing a genome to 100,000 others took <1 min using hash-cgMLST. Hash-cgMLST is an effective surveillance tool for rapidly identifying clusters of related genomes. However, cgMLST/hash-cgMLST generate more false variants than mapping-based approaches. Follow-up mapping-based analyses are likely required to precisely define close genetic relationships.

## Abstract (precis)

Pathogen whole-genome sequencing has huge potential as a tool to better understand infection transmission. However, rapidly identifying closely related genomes among a background of thousands of other genomes is challenging. Here, we describe a refinement to core genome multilocus sequence typing (cgMLST) in which alleles at each gene are reproducibly converted to a unique hash, or short string of letters (hash-cgMLST).

## INTRODUCTION

The rapid development of pathogen whole-genome sequencing offers huge potential for better understanding the epidemiology of many infections. When trying to intervene to stop transmission, it is often important to identify the most closely genetically related organisms already sequenced, as these represent potential recent sources of infection or cases that share an infection source. However, the rapidly growing scale of data generated makes identifying these closely related genomes among a background of many thousands of other genomes very challenging.

Three main approaches can be taken to identify closely related genomes. Comparing single nucleotide polymorphisms (SNPs) identified following mapping to a reference genome offers high precision (1), but despite efforts to optimize computational approaches (2) it is relatively slow. In contrast, k-mer-based approaches based on hash algorithms, e.g., MASH (3) and PopPUNK (4), are fast, but the inherent and unstructured dimensionality reduction (e.g., summarizing the whole genome as 500 hash strings selected on the basis of sorted hash strings) can reduce precision in fine-scale transmission analyses. Core genome multilocus sequencing typing (cgMLST) (5) potentially provides a solution; genomes are summarized as a list of ∼2,000 to 3,000 numbers, with each number representing the unique sequence of each core gene, i.e., structured dimensionality reduction. This summary enables more rapid comparisons, as, taking the example of Clostridium difficile, only 2,270 gene allele numbers need be compared (6), rather than having to compare 4.3 million base pairs of sequence data for SNPs. A drawback of cgMLST as described to date is that it requires a centralized database of alleles of each gene to be maintained so cgMLST profiles generated by different laboratories are comparable. This centralized support can potentially be provided by academic, public health, or commercial organizations, but any given scheme’s sustainability is potentially limited by the funding available to support it. Additionally, for some pathogens, including C. difficile, several competing cgMLST/whole-genome MLST schemes (e.g., Enterobase [University of Warwick, UK], the cgMST.org Nomenclature Server [Ridom GmbH, Germany], and BioNumerics [bioMérieux, France]) containing different genes and profiles have been developed; the latter two are associated with commercial platforms for processing sequencing data.

We therefore propose an alternative to cgMLST as described to date. Instead of maintaining a database of alleles, each allele is reproducibly converted to a unique hash, or short string of letters. This compresses each item of identical data to the same smaller representation, based on the sequence of an allele alone. Therefore, this process can be undertaken independently in different laboratories without the need to maintain or subscribe to a central database, but it still generates summary data in a reproducible form that can be exchanged by laboratories. This distributed approach avoids the potentially costly need to maintain a central database.

This study has two main aims. The first is to demonstrate an implementation of hash-based cgMLST and to test whether hash-cgMLST profiles can be compared without a significant performance penalty compared to standard cgMLST; the second is to test the reproducibility and discriminatory power of cgMLST compared to SNP-based typing. The discriminatory power of cgMLST has been previously explored (for examples, see references 6–9); however, how cgMLST gene differences relate to SNP distances has not been comprehensively assessed. Instead, it is postulated that small numbers of SNPs are likely to fall in different genes, and so SNP distances and gene differences are likely to be similar for closely related isolates. We evaluate the extent to which this assumption holds. Related to this, only limited assessments of the reproducibility of cgMLST have been undertaken. The largest study to date involved the same Staphylococcus aureus DNA from 20 isolates undergoing sequencing in 5 laboratories (10). In this setting, in 80 comparisons (i.e., 20 sequences from 4 laboratories compared with the baseline laboratory), only 3 false gene differences were identified. We investigate whether these results can be replicated in C. difficile.

## MATERIALS AND METHODS

### Hash-cgMLST.

Using the cgMLST scheme of Bletz et al. (6), the first allele for each of the 2,270 genes was used to create a BLAST search query. Following previous descriptions (6, 10), BLAST searches for each gene required a 90% identity match, a matched length ≥99% of the query length, and the matched gene to be free from ambiguous characters or premature truncation. To avoid apparent truncated genes arising from misassembly, we checked the number of stop codons in the gene sequence and only retained matches with a single stop codon. To avoid truncation arising from contig breaks, we ensured that BLAST matches included the start and end of the query sequence. Other BLAST search parameters were as follows: “evalue=0.01, word_size=11, penalty=-1, reward=1, gapopen=5, gapextend=2.” The resulting genes were either matched to the database available at cgMLST.org, i.e., standard cgMLST, or hashed using an md5 algorithm to create a 32-character hexadecimal string. Deletions relative to the search query, represented by dashes in the matched gene sequence, were removed prior to generating the hash. This avoids false differences introduced by locally variable placement of these deletions introduced by BLAST. The resulting cgMLST and hash-cgMLST profiles were saved as JavaScript Object Notation (JSON) files, i.e., a format that could readily be exchanged between laboratories. Where no BLAST match was found for a gene in the scheme, an empty value was recorded and that gene excluded in pairwise comparisons.

The choice of md5 hash provides 16^32^, i.e., 3.4 × 10^38^, possible hashes. There is a theoretical chance of hash collisions, i.e., different sequences resulting in the same hash, but as the number of viable sequences for each gene in cgMLST databases is typically only tens to hundreds, this is very unlikely. Importantly, if a hash collision occurred, this would result in genomes appearing falsely more similar, rather than in falsely excluding potential transmission.

### Sequence data.

During whole-genome sequencing of C. difficile undertaken in Oxford and Leeds (UK), we have routinely resequenced a subset of isolates as part of our internal quality assurance. We searched our database for isolates sequenced more than once. For a subset of these replicate sequences, the same extracted DNA was used to generate both sequences; for the remainder, it was not documented in our laboratory information management system whether the same DNA extract was resequenced or whether a fresh DNA extract was made from the same frozen isolate (Table S1). Paired-end sequence data for both types of replicate were generated using Illumina technology, including various iterations of the HiSeq and MiSeq platforms, with read lengths ranging from 100 to 150 bp for the majority of the sequences (two 50-bp sequences were also included).

To compare the discriminatory power of hash-cgMLST compared to SNP-based typing, we processed 973 genomes from a previously published study of consecutive C. difficile infections over 1 year in six English hospitals using our hash-cgMLST and SNP pipelines (11).

### Bioinformatic processing.

For hash-cgMLST typing, raw sequence data underwent adapter trimming and quality trimming using bbduk.sh from the BBMap package (version 38.32) (12). Stringent quality trimming was applied following Mellmann et al. (10); both the left and right ends of each read were trimmed to a Q30 threshold (using BBDuk parameters “ktrim=r k=23 mink=11 hdist=1 tpe tbo qtrim=rl trimq=30”). Following this, the number of bases remaining in the trimmed reads was divided by the length of the 630 reference genome (13), 4,290,252 bp, to provide the mean high-quality coverage; this was required to be ≥50× for a sequence to be included in the study. Appropriate quality trimming and adapter removal were confirmed using FastQC (14). To check for contamination with non-C. difficile DNA, the species origin of sequence reads was classified using Kraken2 (15) and the MiniKraken2_v1 database (built from the RefSeq bacteria, archaea, and viral libraries).

Following Bletz et al. (6), reads were *de novo* assembled using SPAdes (version 3.11.1) (16), with the “-careful” flag to reduce misassembly, using Burrows-Wheeler Aligner (BWA)-based mapping to confirm variants. Assembly quality metrics were obtained using the stats.sh script from BBMap (12). Samples with assembly sizes (base pairs in contigs) of >10% more or less than the median size were rejected. We also tested performance using SPAdes with an additional flag, “-only-assembler,” to disable the SPAdes internal read correction procedure. As an additional comparison, reads were also *de novo* assembled using SKESA (version 2.3) (17) with default settings.

Reads (without stringent quality trimming) were also mapped to the 630 reference genome as described previously (1, 11, 18), using stampy (19) for mapping and mpileup (20) for variant calling, followed by quality filtering of variants. Variant calls were required to have a quality score of ≥30, be homozygous under a diploid model, be supported by ≥5 high quality reads (including ≥1 read in each direction and a consensus of ≥90% of bases), and not be within a repetitive region of the genome. See https://github.com/oxfordmmm/CompassCompact for example implementation. For inclusion, ≥70% of the reference genome needed to be called in the consensus sequence. Bases in the consensus sequence not passing quality filtering were denoted N rather than A, C, G, or T.

The bioinformatic pipelines used in this study for assembly and hash-cgMLST were written as Nextflow workflows (21) and can be found at https://github.com/davideyre/hash-cgmlst. Information on required dependencies and system requirements is provided in the repository readme file.

### Analysis.

Sequences meeting all quality thresholds (high-quality average coverage, assembly size, and proportion of reference genome called) were compared. For replicate sequences, when an isolate had been sequenced more than twice, a random sequence was chosen as the baseline sequence to which all other sequences from the same isolate were compared in order to avoid multiple counting.

Pairwise observed SNP differences between replicates and recombination-corrected SNP differences between other C. difficile genomes were obtained using Python scripts, PhyML (22), and ClonalFrameML (23), as previously described (11) (https://github.com/davideyre/runListCompare). Whole-genome alignments were used as input for PhyML. Invariant sites, i.e., those called as the same base as the reference or as an unknown base (N) across all genomes were set to be the same base as the reference for computational efficiency, given that there was no evidence of variation at these sites. All other sites had evidence of variation in at least one genome and were included unchanged, including any genomes with an N at that site. The maximum likelihood approach taken accounts for the uncertainty in the phylogeny arising from some genomes having an N called at some variable sites.

The number of cgMLST locus differences and number of loci compared were obtained using Python (https://github.com/davideyre/hash-cgmlst). Where no BLAST match was found for a gene in either (or both) of the genomes in a pairwise comparison, this was not counted toward the total number of cgMLST gene differences.

### Data availability.

Sequence Read Archive (SRA) accession numbers for analyzed replicate genomes are provided in Table S1, with explanatory notes in the accompanying legend. Data for the 973 genomes from six English hospitals can be found under NCBI BioProject accession number PRJNA369188.

## RESULTS

Hash-cgMLST provided the same results as standard cgMLST with a minimal performance penalty. Results are presented throughout using pairwise core-gene differences generated with hash-cgMLST, as these were identical to standard cgMLST gene differences if novel alleles were accounted for.

### Comparison of hash-cgMLST and SNP typing performance in replicate sequences.

A total of 374 sequences from 104 isolates passed all quality checks and were available for comparison to investigate the reproducibility of sequencing followed by cgMLST for C. difficile transmission analyses. A median of 2 (interquartile range [IQR], 2 to 3; range, 2 to 27) sequences were available per isolate. Comparing replicate sequences with a randomly selected baseline sequence for each isolate yielded 272 comparisons for analysis.

**FIG 1:**
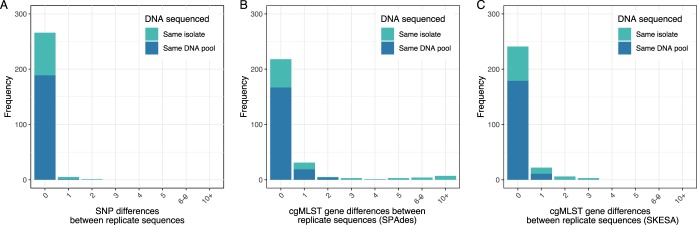
Observed differences using SNP typing (panel A) and hash-cgMLST based on SPAdes (panel B) and SKESA (panel C) assemblies in 272 replicate sequence pairs. With perfect sequencing, no variants would be expected between pairs of sequences from the same isolate. Pairs of sequences known to have been obtained from the same pool of DNA are shown in dark blue. Where information was unavailable on whether the same pool of DNA was used or a fresh DNA extract was made from the same isolate, this is shown in light blue.

With perfect sequencing, no variants would be expected between pairs of sequences from the same isolate (replicate pairs). Using reference-based mapping and variant calling, there were 0 SNPs between 262 (96%) replicate pairs, 1 SNP between 5 (2%) pairs, and 2 SNPs between 1 (<1%) pair, i.e., a mean of 0.026 SNPs per pair, which equates to 1 false SNP call per 39 sequences (Fig. 1A). Based on the rate of C. difficile evolution and the extent of within-host genetic diversity, ≤2 SNPs are expected between >95% of cases related by recent transmission (1); it is therefore unlikely that transmission would be falsely excluded on the basis of the error rates seen.

Using either hash-cgMLST or standard cgMLST following assembly using SPAdes, 218 (80%) replicate pairs had zero gene differences, 31 (11%) pairs had 1 difference, 5 (2%) pairs had 2 differences, and 18 (7%) pairs had >2 differences, with a mean of 0.64 false gene differences per genome (Fig. 1B) (test for symmetry considering 0, 1, 2, and >2 SNPs or gene differences; *P* = 0.004). Applying a threshold of >2 gene differences to rule out transmission (by analogy with SNP-based metrics [1, 6]), the observed error rate would result in 6.6% (95% binomial confidence interval [CI], 4.0 to 10.3%) of transmission pairs being falsely excluded. Restriction to the subset of sequences for which sequencing was known to have been undertaken from the same pool of extracted DNA produced fewer gene differences (Fig. 1). Of 190 pairs, 189 (>99%) had 0 SNPs, and 1 (<1%) pair had 1 SNP. Based on cgMLST, 167 (88%) pairs had 0 gene differences, 19 (10%) had 1 difference, 4 (2%) had 2 differences, and none had >2 differences.

### Predictors of false cgMLST gene differences.

**FIG 2:**
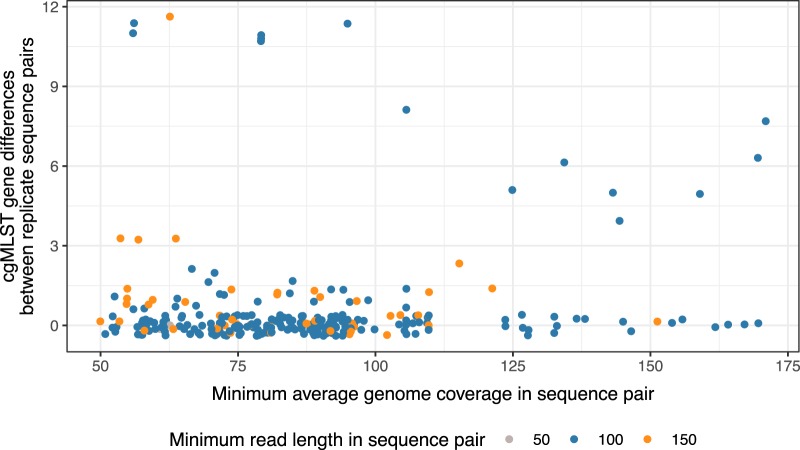
Relationship between hash-cgMLST gene differences in replicate sequence pairs and average genome coverage and read length. Jitter applied to points to assist visualization. SPAdes with the “-careful” flag was used to generate assemblies.

The observation of greater differences between replicates when restricted to variation in the 2,270 core genes versus considering SNPs across the whole genome is potentially counterintuitive. However, it should be remembered that the whole-genome SNP approach depends on a different bioinformatic approach with sophisticated per-variant quality filtering, whereas cgMLST is based on *de novo* assembly with more limited quality filtering. We therefore investigated potential predictors of false cgMLST gene differences using the hash-cgMLST algorithm (potential predictors were identical to those in the standard cgMLST approach) to see if filtering could be improved. Although we had already restricted our analysis to only include sequences with a mean genome coverage of >50×, we investigated whether a more stringent threshold would improve performance (Fig. 2). There was no evidence that increased coverage was associated with fewer cgMLST gene differences (Spearman’s rho = −0.04; *P* = 0.43). There were only 2 sequences in the data set with 50-bp reads; the remainder had 100- or 150-bp reads. In total, 14/222 (6%) sequence pairs in which the minimum sequence length was 100 bp contained >2 gene differences, compared to 4/48 (8%) in pairs with both 150-bp reads (exact *P* = 0.54).

**FIG 3:**
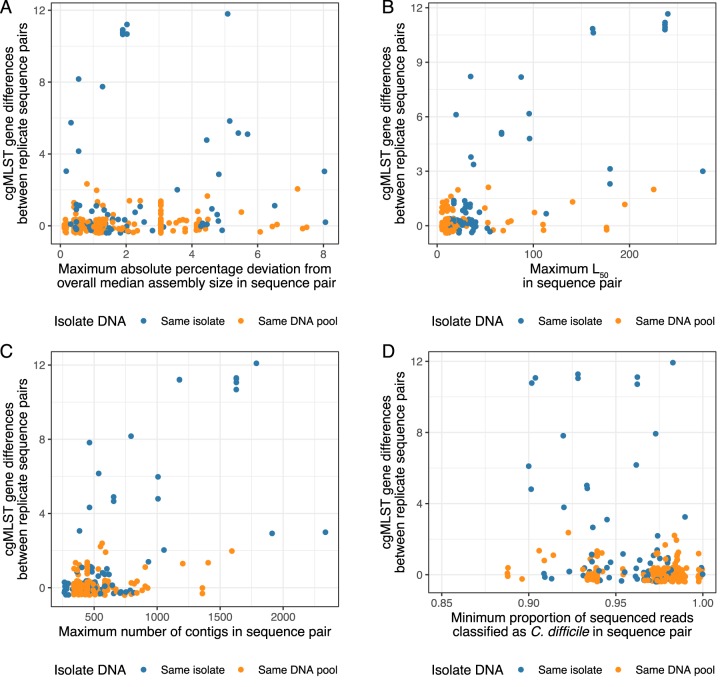
Relationship between hash-cgMLST gene differences in replicate sequence pairs and *de novo* assembly quality metrics (A to C) and Kraken2 read classification (D). Jitter applied to points to assist visualization. One point is omitted from Fig. 3D for ease of visualization with the proportion of reads classified as C. difficile (0.64) and 0 gene differences. SPAdes with the “-careful” flag was used to generate assemblies.

The relationship between cgMLST gene differences and *de novo* assembly quality metrics is shown in Fig. 3A to C. Given the filtering applied, there was still an association between the number of false gene differences and the maximum absolute percentage deviation from the overall median assembly size (4,165,590 bp) within each replicate pair (which was constrained to be ≤10% for inclusion in the analysis) (Spearman’s rho = 0.21; *P* < 0.001; Fig. 3A, with both small and large assemblies contributing to this effect). The *L*_50_ value describes the minimum number of contigs required to achieve 50% of the assembly size, with higher values representing more fragmented lower quality assemblies. Higher *L*_50_ values were associated with greater rates of false gene differences (Spearman’s rho = 0.37; *P* < 0.001). A total of 9 (2%) of 257 pairs with both *L*_50_ values of ≤125 had >2 false gene differences, compared to 9/15 (60%) with one or more sequences with an *L*_50_ value of >125 (Fig. 3B). Another measure of assembly fragmentation is the total number of contigs; higher numbers of contigs were also associated with greater false gene differences (Spearman’s rho = 0.31; *P* < 0.001; Fig. 3C).

Figure 3D shows the impact of the proportion of reads classified as C. difficile by Kraken2 on cgMLST gene differences. Within the data set, there was no evidence of significant contamination with a bacterial species other than C. difficile, and the most common species in all samples was C. difficile. However, the proportion of reads that could not be classified at all varied from 0 to 11% between sequences, with the exception of one replicate pair (36% and 24%). Higher rates of unclassified sequences were associated with higher false gene differences but without any clear separation of the data on this basis (Spearman’s rho = −0.23; *P* < 0.001).

### Distribution of cgMLST gene differences in replicate sequences.

The gene differences observed between replicate sequences disproportionately affected a small number of genes (Table S2). Only 82 (4%) of 2,270 genes contained differences within the replicate sequences. To avoid multiple counting, we evaluated the number of isolates that contained at least a pair of replicates with gene differences. A total of 16 genes contained differences in two or more isolates’ replicates and, of these, 15 were due to the same nucleotide differing in all replicate pairs. The reproducible location of the differences observed for a given gene across different isolates is compatible with consistent misassembly (Table S2). If the 15 genes with identical gene differences affecting ≥2 isolates were excluded, the number out of the 272 replicate pairs with 0 gene differences increased from 218 (80%) to 236 (87%), and the number of pairs with >2 gene differences reduced from 18 (7%) to 14 (5%). (Fig. S1B). Using the full 2,270-gene set and disabling SPAdes internal read correction resulted in fewer false gene differences, namely, 0 differences in 236 (87%) pairs and >2 differences in 14 (5%) (Fig. S1C).

### Alternative assembler (SKESA).

Use of SKESA in place of SPAdes as the assembler used for hash-cgMLST resulted in fewer differences between replicate pairs (Fig. 1C), namely, 241 (89%) pairs had 0 differences, 22 (8%) pairs had 1 difference, 6 (2%) pairs had 2 differences, and 3 (1%) pairs had 3 differences. This equates to 0.16 false gene differences per replicate pair sequenced. The median number of genes compared between replicate pairs was 2,225 (IQR, 2,187 to 2,235) using SKESA and 2,227 (IQR, 2,205 to 2,242) using SPAdes out of a possible maximum of 2,270 genes.

### Benchmarking.

Samples were processed in parallel, with each sample using a single core from an Intel Xeon Gold 6150 2.70-GHz 18-core central processing unit (CPU). For a single sample, the median (IQR) time to undertake quality control and read filtering was 3.6 (2.7 to 4.9) minutes and 27.4 (19.6 to 35.4) minutes, respectively, to generate an assembly using Spades with read error correction and 16.3 (12.1 to 21.5) minutes without; SKESA took 19.4 (15.5 to 24.3) minutes. Creating a hash-cgMLST profile from the assemblies took 44.1 (43.5 to 44.9) seconds. After making hash-cgMLST profile files, comparing a single genome to 100,000 others using a single CPU core took 40.4 s. In contrast, 100,000 comparisons using a standard cgMLST approach took marginally less time—38.7 s—after loading the profiles into memory.

cgMLST profiles can also be rapidly compared using a laptop or desktop; for example, using one core of an Intel i7 2.6-Ghz laptop processor, comparing the 973 samples from the six hospitals study required 467 Mb of memory and took 236 s for 472,879 comparisons, i.e., 49.9 s per 100,000 comparisons. Using the same laptop, creating hash-cgMLST profiles from existing assemblies typically took ∼40 s and required <100 Mb of memory.

### Comparison of hash-cgMLST and SNP typing in data from six English hospitals.

**FIG 4:**
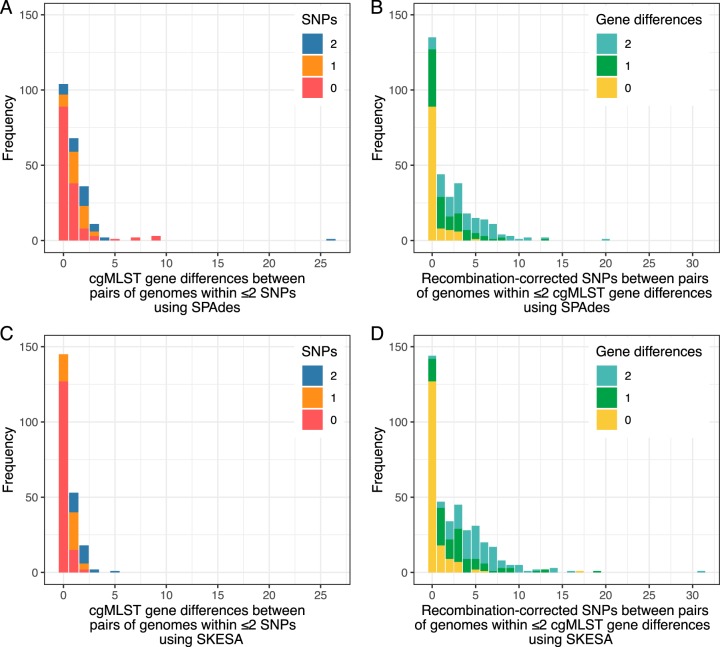
Relationship between hash-cgMLST gene differences and SNPS in C. difficile genomes from consecutive infections in six English hospitals. (A) Distribution of hash-cgMLST gene differences between pairs of genomes within ≤2 SNPs. (B) Distribution of SNPs within pairs of genomes within ≤2 gene differences. Panels A and B were generated using SPAdes assemblies with the “-careful -only-assembler” flags. (C and D) The same analysis using the SKESA assembler.

We analyzed 973 genomes from a previous study of C. difficile transmission in six English hospitals (11). Of these, 56 failed the assembly size threshold and 20 failed the coverage threshold (one of these also failed the assembly threshold), leaving 898 (92%) genomes for analysis. We considered all pairs of genomes within ≤2 SNPs and used SPAdes (with the -only-assembler flag) or SKESA assemblies to test the extent to which the numbers of hash-cgMLST gene differences followed the number of SNPs (Fig. 4A and C). Of 412 pairs of sequences differing by ≤2 SNPs, according to analysis using SPAdes assemblies, 376 (91%) were within ≤2 gene differences, 30 (7%) had 3 differences, and 16 (4%) had ≥4 differences; according to analysis using SKESA assemblies, 406 (99%) had ≤2 gene differences, and the remainder all had ≤5 differences. The median number of genes called in each pair was 2,143 (IQR, 2,084 to 2,191) using SPAdes and 2,003 (IQR, 1,891 to 2,110) using SKESA.

To achieve ≥99% sensitivity for identifying genomes within ≤2 SNPs required a threshold of ≤9 gene differences using SPAdes and ≤3 gene differences using SKESA, with associated positive predictive values (PPVs) of 11% (410/3,720) and 38% (410/1,092), respectively. Specificity was >99% with both assemblers (399,031/402,341 and 401,659/402,341, respectively).

We also considered the distribution of recombination-corrected SNPs within pairs of genomes with ≤2 gene differences using hash-cgMLST. Following assembly with SPAdes, of 590 pairs of genomes, 376 (64%) were within ≤2 SNPs, with the maximum number of SNPs observed being 20 (Fig. 4B). Using SKESA analysis of 749 genome pairs, 406 (54%) were within ≤2 SNPs (Fig. 4D).

## DISCUSSION

Here, we present the concept of hash-cgMLST as a tool for rapid comparison of bacterial sequencing data. This is a significant development from standard cgMLST approaches, as it removes the need for a central database of alleles. Such databases require resource-intensive curation to ensure they are maintained to a high standard. Additionally, allele numbering is currently done consecutively in a single location, which is problematic with large data sets that span many laboratories; hashes also overcome this limitation. We also provide the code to run the algorithms developed.

This article also highlights important limitations of common implementations of cgMLST as a tool for high-resolution outbreak detection. Stringent filtering done on the basis of mapped data allows the number of false variant calls to be controlled; here, we obtained around 1 false SNP for every 39 genomes sequenced. In contrast, fine-grained per-base quality control is typically not implemented in studies using *de novo* assembly tools. Using SPAdes, we observed a mean of 0.64 false gene differences per replicate genome pair. The alternative assembler tested, SKESA, was able to better control false gene differences, with 0.16 per replicate pair, i.e., 1 error per every 6.3 genomes sequenced. The higher rates of false variation observed using cgMLST/hash-cgMLST led to the counterintuitive observation in some samples of more differences when comparing 2,270 genes than when comparing the whole genome. It should be noted that undertaking SNP-based analyses from alignments of *de novo* assemblies without further filtering of variants would be similarly affected. These errors can be reduced by ensuring that the assemblies studied are of high quality. Our data suggest that the previously described read quality trimming and filtering based on assembly sizes (6, 10) could be further improved by also only analyzing samples with an *L*_50_ value of less than ∼125. However, this stringent filtering would have resulted in 30% of the previously published data set studied being unavailable for analysis, calling its practicability into question.

Although our approach does not depend on a database of alleles, it is dependent on the development of a high-quality cgMLST scheme, i.e., appropriate identification of core genes based on a large and diverse collection of genomes and careful selection of problematic genes for exclusion. Despite such an approach being taken in developing the C. difficile cgMLST scheme used, we show that removing a small number of genes from this cgMLST scheme would likely improve performance if using SPAdes assemblies, as a small subset of genes contained higher numbers of false gene differences (Table S2, Fig. S1). This highlights the importance of assessing the performance of each cgMLST scheme created on a per-species and per-scheme basis using appropriate test data sets that include replicate and closely related sequences.

Many of the apparent errors seen in replicate pairs appear to arise from misassembly. SPAdes-based read correction did not improve accuracy and instead resulted in more, rather than fewer, differences between replicate pairs. Use of an alternative assembler, SKESA (17), reduced the number of replicate pairs with >2 differences to just 1%, with a minimal reduction in the number of genes compared between replicate pairs (median of 2,225, compared to 2,227 with SPAdes). The reduction in genes compared was greater in the clinical data set analyzed (medians of 2,143 and 2,003), but this reduced discriminatory power for transmission studies will usually be more than offset by reduced error rates (and therefore reductions in erroneous exclusion of transmission).

Our data also highlight that extrapolating the ≤2-SNP threshold for identifying genetically plausible transmission events to two (or three [6]) gene differences may be inappropriate, depending on the choice of assembler and settings. Using SPAdes, 4% of pairs of samples within ≤2 SNPs were >3 genes different by cgMLST, whereas with SKESA this was only 1%. For public health applications optimized to identify potential transmission, to be ≥99% sure of not missing pairs of sequences within ≤2 SNPs, a threshold of ≤9 gene differences was needed for SPAdes assemblies and ≤3 differences with SKESA. However, these thresholds for SPAdes resulted in around 8 genome pairs that were >2 recombination-corrected SNPs apart being identified for every 1 pair within ≤2 SNPs (PPV, 11%) and 1.6 pairs that were >2 SNPs apart for every pair within ≤2 SNPs using SKESA (PPV, 38%). In this scenario, further SNP-based analysis based on mapping and filtered variant calling is likely to be required to determine which genomes are potentially related by recent transmission and which are not. In other cases, larger numbers of SNPs than gene differences were observed (Fig. 4B and D), which may arise from SNPs outside core genes, SNPs in uncalled genes, and imperfect correction of recombination events.

Hash-cgMLST allowed rapid comparison of many thousands of bacterial genomes within seconds, using a relatively unoptimized Python script running on a single laptop or server CPU core. As comparisons with other genomes can be easily divided into independent parts, this task is readily parallelizable. Using hash-cgMLST, it is therefore potentially possible to compare each new sequence generated with millions of previous sequences. The summaries of each genome produced a roughly 130-kb JSON file, which is readily exchangeable between laboratories and could potentially be hosted alongside raw reads in sequence read archives. As such, each laboratory could maintain its own database of hash-cgMLST profiles and distances, as well as this information potentially being usefully provided as part of future web-based services based on publicly available data. Although, without further refinements, hash-cgMLST may not allow high-precision fine-scaled transmission studies, it has the potential to dramatically reduce the search space for closely related genomes, which can then be followed by more precise SNP-based analyses on a much smaller subset of genomes.

Using SPAdes, we observed a higher rate of “false” gene differences between genomes in which the sequences were potentially generated from separate DNA extractions of the same isolates compared to that in genomes obtained from the same DNA extraction. It is therefore plausible that the differences observed represent true differences but also a form of variation that is much faster and more erratic than mutation/recombination rates based on filtered SNPs. The erratic nature of the variation observed is unlikely to be informative about recent transmission. We also did not see these differences to the same extent using an alternative assembler, SKESA.

This study is potentially limited by not being an exhaustive investigation of all the potential options for assembly and for filtering *de novo* assembly data; in particular, further filtering of variants based on mapping reads back to assemblies, e.g., as done by Enterobase (24), may improve precision. Although we used Kraken2 to search for contamination with DNA from other species, contamination with C. difficile DNA from other concurrently processed samples may be an important contributor to some of the differences seen with hash-cgMLST, whereas resulting mixed calls can be filtered using mapped data.

In conclusion, appropriately quality controlled cgMLST can identify clusters of related genomes rapidly and is an appropriate tool for surveillance and reducing the search space in outbreaks. The SKESA assembler, compared to SPAdes, was associated with lower rates of gene differences between replicate sequences and, when used for hash-cgMLST, more closely matched the number of SNPs between closely related samples. The approach we describe has potential to be deployed across a range of pathogens, including those where linkage across time and wide geographic space, i.e., cases involving very large sequencing data sets, may help resolve sources and routes of transmission, such as for foodborne infections. Refined variant calling based on mapping is likely required to precisely define close genetic relationships. This study highlights the need for detailed quality assurance to determine the performance of algorithms used for comparing genomes. Our hash-cgMLST implementation is freely available and provides an effective database-free approach to cgMLST.

## Supplementary Material

Supplemental file 1

Supplemental file 2

Supplemental file 3

## References

[1] Eyre DW, Cule ML, Wilson DJ, Griffiths D, Vaughan A, O’Connor L, Ip CLC, Golubchik T, Batty EM, Finney JM, Wyllie DH, Didelot X, Piazza P, Bowden R, Dingle KE, Harding RM, Crook DW, Wilcox MH, Peto TEA, Walker AS 2013 Diverse sources of *C. difficile* infection identified on whole-genome sequencing. N Engl J Med 369:1195–1205. doi:10.1056/NEJMoa1216064. 24066741 PMC3868928

[2] Mazariegos-Canellas O, Do T, Peto T, Eyre DW, Underwood A, Crook D, Wyllie DH 2017 BugMat and FindNeighbour: command line and server applications for investigating bacterial relatedness. BMC Bioinformatics 18:477. doi:10.1186/s12859-017-1907-2. 29132318 PMC5683244

[3] Ondov BD, Treangen TJ, Melsted P, Mallonee AB, Bergman NH, Koren S, Phillippy AM 2016 Mash: fast genome and metagenome distance estimation using MinHash. Genome Biol 17:132. doi:10.1186/s13059-016-0997-x. 27323842 PMC4915045

[4] Lees JA, Harris SR, Tonkin-Hill G, Gladstone RA, Lo SW, Weiser JN, Corander J, Bentley SD, Croucher NJ 2019 Fast and flexible bacterial genomic epidemiology with PopPUNK. Genome Res 29:304–316. doi:10.1101/gr.241455.118. 30679308 PMC6360808

[5] Maiden MC, van Rensburg MJ, Bray JE, Earle SG, Ford SA, Jolley KA, McCarthy ND 2013 MLST revisited: the gene-by-gene approach to bacterial genomics. Nat Rev Microbiol 11:728. doi:10.1038/nrmicro3093. 23979428 PMC3980634

[6] Bletz S, Janezic S, Harmsen D, Rupnik M, Mellmann A 2018 Defining and evaluating a core genome multilocus sequence typing scheme for genome-wide typing of *Clostridium difficile*. J Clin Microbiol 56:e01987-17. doi:10.1128/JCM.01987-17. 29618503 PMC5971537

[7] Ruppitsch W, Pietzka A, Prior K, Bletz S, Fernandez H, Allerberger F, Harmsen D, Mellmann A 2015 Defining and evaluating a core genome multilocus sequence typing scheme for whole-genome sequence-based typing of *Listeria monocytogenes*. J Clin Microbiol 53:2869–2876. doi:10.1128/JCM.01193-15. 26135865 PMC4540939

[8] de Been M, Pinholt M, Top J, Bletz S, Mellmann A, van Schaik W, Brouwer E, Rogers M, Kraat Y, Bonten M, Corander J, Westh H, Harmsen D, Willems RJ 2015 Core genome multilocus sequence typing scheme for high-resolution typing of *Enterococcus faecium*. J Clin Microbiol 53:3788–3797. doi:10.1128/JCM.01946-15. 26400782 PMC4652124

[9] Cody AJ, Bray JE, Jolley KA, McCarthy ND, Maiden MC 2017 Core genome multilocus sequence typing scheme for stable, comparative analyses of *Campylobacter jejuni* and *C. coli* human disease isolates. J Clin Microbiol 55:2086–2097. doi:10.1128/JCM.00080-17. 28446571 PMC5483910

[10] Mellmann A, Andersen P, Bletz S, Friedrich AW, Kohl TA, Lilje B, Niemann S, Prior K, Rossen JW, Harmsen D 2017 High interlaboratory reproducibility and accuracy of next-generation-sequencing-based bacterial genotyping in a ring trial. J Clin Microbiol 55:908–913. doi:10.1128/JCM.02242-16. 28053217 PMC5328459

[11] Eyre DW, Fawley WN, Rajgopal A, Settle C, Mortimer K, Goldenberg SD, Dawson S, Crook DW, Peto TE, Walker SA, Wilcox MH 2017 Comparison of control of *Clostridium difficile* infection in six English hospitals using whole-genome sequencing. Clin Infect Dis 65:433–441. doi:10.1093/cid/cix338. 28575285 PMC5850028

[12] Bushnell B BBMap. http://sourceforge.net/projects/bbmap/.

[13] Sebaihia M, Wren BW, Mullany P, Fairweather NF, Minton N, Stabler R, Thomson NR, Roberts AP, Cerdeño-Tárraga AM, Wang H, Holden MT, Wright A, Churcher C, Quail MA, Baker S, Bason N, Brooks K, Chillingworth T, Cronin A, Davis P, Dowd L, Fraser A, Feltwell T, Hance Z, Holroyd S, Jagels K, Moule S, Mungall K, Price C, Rabbinowitsch E, Sharp S, Simmonds M, Stevens K, Unwin L, Whithead S, Dupuy B, Dougan G, Barrell B, Parkhill J 2006 The multidrug-resistant human pathogen *Clostridium difficile* has a highly mobile, mosaic genome. Nat Genet 38:779–786. doi:10.1038/ng1830. 16804543

[14] Andrews S, Babraham Bioinformatics. FastQC. https://www.bioinformatics.babraham.ac.uk/projects/fastqc/.

[15] Wood DE, Salzberg SL 2014 Kraken: ultrafast metagenomic sequence classification using exact alignments. Genome Biol 15:R46. doi:10.1186/gb-2014-15-3-r46. 24580807 PMC4053813

[16] Bankevich A, Nurk S, Antipov D, Gurevich AA, Dvorkin M, Kulikov AS, Lesin VM, Nikolenko SI, Pham S, Prjibelski AD, Pyshkin AV, Sirotkin AV, Vyahhi N, Tesler G, Alekseyev MA, Pevzner PA 2012 SPAdes: a new genome assembly algorithm and its applications to single-cell sequencing. J Comput Biol 19:455–477. doi:10.1089/cmb.2012.0021. 22506599 PMC3342519

[17] Souvorov A, Agarwala R, Lipman DJ 2018 SKESA: strategic k-mer extension for scrupulous assemblies. Genome Biol 19:153. doi:10.1186/s13059-018-1540-z. 30286803 PMC6172800

[18] Eyre DW, Davies KA, Davis G, Fawley WN, Dingle KE, Maio N, Karas A, Crook DW, Peto TE, Walker SA, Wilcox MH, EUCLID Study Group. 2018 Two distinct patterns of *Clostridium difficile* diversity across Europe indicates contrasting routes of spread. Clin Infect Dis 67:1035–1044. doi:10.1093/cid/ciy252. 29659747 PMC6137122

[19] Lunter G, Goodson M 2011 Stampy: a statistical algorithm for sensitive and fast mapping of Illumina sequence reads. Genome Res 21:936–939. doi:10.1101/gr.111120.110. 20980556 PMC3106326

[20] Li H, Handsaker B, Wysoker A, Fennell T, Ruan J, Homer N, Marth G, Abecasis G, Durbin R 2009 The Sequence Alignment/Map format and SAMtools. Bioinformatics 25:2078–2079. doi:10.1093/bioinformatics/btp352. 19505943 PMC2723002

[21] Tommaso P, Chatzou M, Floden EW, Barja P, Palumbo E, Notredame C 2017 Nextflow enables reproducible computational workflows. Nat Biotechnol 35:316–319. doi:10.1038/nbt.3820. 28398311

[22] Guindon S, Dufayard J-F, Lefort V, Anisimova M, Hordijk W, Gascuel O 2010 New algorithms and methods to estimate maximum-likelihood phylogenies: assessing the performance of PhyML 3.0. Syst Biol 59:307–321. doi:10.1093/sysbio/syq010. 20525638

[23] Didelot X, Wilson DJ 2015 ClonalFrameML: efficient inference of recombination in whole bacterial genomes. PLoS Comput Biol 11:e1004041. doi:10.1371/journal.pcbi.1004041. 25675341 PMC4326465

[24] Alikhan N-F, Zhou Z, Sergeant MJ, Achtman M 2018 A genomic overview of the population structure of *Salmonella*. PLoS Genet 14:e1007261. doi:10.1371/journal.pgen.1007261. 29621240 PMC5886390

