# Supplementary material for: Hash-Based Core Genome Multilocus Sequence Typing for Clostridium difficile
Source: J Clin Microbiol. 2019 Dec 23;58(1):e01037-19. doi: 10.1128/JCM.01037-19 (PMC6935933; doi:10.1128/JCM.01037-19)

Hash-based core genome multi-locus sequencing typing for *Clostridium difficile*: Supplement

Dr David W Eyre, Prof Tim EA Peto, Prof Derrick W Crook, Prof A Sarah Walker, Prof Mark H Wilcox

Supplementary table legends

Table S1. Replicate sequence identifiers. This table provides details of the sequence identifiers for the replicate sequences studied, including SRA sample identifiers for each sequence. Isolates ending in _p1, _p2, etc indicate that these replicates were made from the same numbered pool of DNA.

Table S2. Genes with gene differences identified amongst replicate sequencing of the same isolates, using assemblies made with SPAdes.

Supplementary figure

Figure S1. Observed differences using hash-cgMLST based on SPAdes assemblies in 272 replicate sequence pairs, before (panel A) and after (panel B) excluding 15 genes with high rates of mis-assembly or without SPAdes read correction (panel C). Pairs of sequences known to have been obtained from the same pool of DNA are shown in dark blue. Where information was unavailable on whether the same pool of DNA was used or a fresh DNA extract was made from the same isolate, this is shown in light blue.

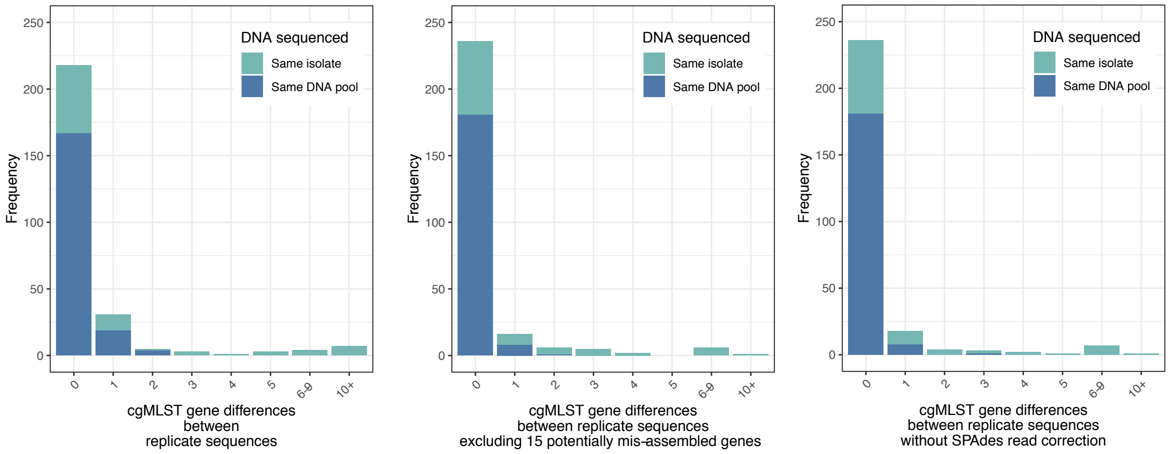

Supplement: Supplemental file 1 [file JCM.01037-19-s0001.pdf]
